# Analysis of Transposable Elements in *Coccidioides* Species

**DOI:** 10.3390/jof4010013

**Published:** 2018-01-19

**Authors:** Theo N. Kirkland, Anna Muszewska, Jason E. Stajich

**Affiliations:** 1 Departments of Pathology and Medicine, School of Medicine, University of California, San Diego, CA 92037, USA; 2 Institute of Biochemistry and Biophysics, Polish Academy of Sciences, 02-106 Warsaw, Poland; musze@ibb.waw.pl; 3 Department of Microbiology and Plant Pathology, Institute for Integrative Genome Biology, University of California-Riverside, Riverside, CA 92521, USA; jason.stajich@ucr.edu

**Keywords:** fungus, *Coccidioides* spp., genomics, transcriptome, transposable elements

## Abstract

*Coccidioides immitis* and *C. posadasii* are primary pathogenic fungi that cause disease in immunologically-normal animals and people. The organism is found exclusively in arid regions of the Southwestern United States, Mexico, and South America, but not in other parts of the world. This study is a detailed analysis of the transposable elements (TE) in *Coccidioides* spp. As is common in most fungi, Class I and Class II transposons were identified and the LTR *Gypsy* superfamily is the most common. The minority of *Coccidioides Gypsy* transposons contained regions highly homologous to polyprotein domains. Phylogenetic analysis of the integrase and reverse transcriptase sequences revealed that many, but not all, of the *Gypsy* reverse transcriptase and integrase domains clustered by species suggesting extensive transposition after speciation of the two *Coccidiodies* spp. The TEs were clustered and the distribution is enriched for the ends on contigs. Analysis of gene expression data from *C. immitis* found that protein-coding genes within 1 kB of *hAT* or *Gypsy* TEs were poorly expressed. The expression of *C. posadasii* genes within 1 kB of *Gypsy* TEs was also significantly lower compared to all genes but the difference in expression was smaller than *C. immitis*. *C. posadasii* orthologs of *C. immitis Gyspsy*-associated genes were also likely to be TE-associated. In both *C. immitis* and *C. posadasii* the TEs were preferentially associated with genes annotated with protein kinase gene ontology terms. These observations suggest that TE may play a role in influencing gene expression in *Coccidioides* spp. Our hope is that these bioinformatic studies of the potential TE influence on expression and evolution of *Coccidioides* will prompt the development of testable hypotheses to better understand the role of TEs in the biology and gene regulation of *Coccidioides* spp.

## 1. Introduction

We are reporting an analysis of the predicted transposable elements (TE) in the pathogenic fungi *Coccidioides immitis* and *Coccidioides posadasii* and the potential relationship of TEs with gene expression. *Coccidioides* spp. are found in the soil and associated with kangaroo rats in the desert regions of the southwestern US, Mexico, and Central and South America [1]. There are two species, *C. immitis* and *C. posadasii* that have been identified by DNA polymorphism and genome sequencing [2,3]. The two species are morphologically indistinguishable. *Coccidioides* spp. are haploid fungi and lack a described sexual phase although there is molecular evidence for recombination and genes coding for mating type loci has been identified [3,4,5]. These fungi have unusual life stages where they form spores within mycelia and differentiate into spherules, a rarely observed morphology in fungi, once inside a mammalian host. *Coccidioides* spp. grow in desert soil and the arthroconidia developed from desiccated hyphae when dispersed by the wind can cause disease in mammals, including human beings [6]. Like the other primary pathogenic fungi, *Coccidioides* spp. are dimorphic; in the mammalian host arthroconidia differentiate to spherules which mature by isotropic growth into large structures that reproduce by releasing endospores. This organism is one of the very few fungi that can cause disease in immunocompetent people. More than 30% of human infections are symptomatic, usually causing respiratory symptoms [7,8]. A significant fraction of infections can disseminate beyond the lungs and cause disseminated disease which can be fatal. Hosts that survive an infection can form granuloma of walled off spherules that lay dormant until the host undergoes an immune deficiency or expires of other causes [8].

There have been a small number of studies of the transcriptome of mycelia (the saprobic form) and spherules (the parasitic form) [9]. These studies used spherules produced in vitro, rather than in animals. In vitro production of spherules involves a defined media, elevated temperature and increased CO_2_ tension compared to the growth of mycelia [10]. The spherules produced in vitro appear identical to those in vivo by microscopy, but it is not currently known whether these express a similar gene expression program.

These transcription studies identified 13–22% of *Coccidioides* spp. genes that were up-regulated as mycelia differentiate into spherules [11]. One study found that transcripts of heat shock protein 30, polysaccharide deacetylase (Arp2/3 complex), spherule outer-wall glycoprotein, and others were up-regulated [9]. One hundred fifty genes were up-regulated in both studies [12]. These include transcripts of 4-hydroxyphenylpyruvate dioxygenase (a gene that is required for dimorphism in many fungi) [13], polysaccharide deacetylase (Arp2/3 complex), β-(1,4)-amylase, and many other genes in metabolic pathways of complex carbohydrate remodeling. Keto-reductase, and the major facilitator super family transporter family genes were also up-regulated. Several gene families, including some members of the protein kinase family, were down-regulated in spherules [11].

Transposable elements (TEs) are ubiquitous mobile genetic elements found in all eukaryotic genomes [14,15]. TEs were initially thought to have no function (junk DNA), but more recently have been found to be important in genome evolution, gene duplication and a variety of epigenetic phenomena, including influencing expression of a variety protein coding genes and transcriptional response to stresses [16,17,18]. In some eukaryotes, including insects, vertebrates and especially plants more than half of the genome consists of transposons [16,19]. The fraction of TEs in most fungal genomes varies from 3–20% [20]. However, in extreme cases such as *Blumeria graminis*, 85% of the genes are TEs [21].

Most TE have been classified into Class I and Class II. Class I TEs are retrotransposons that require an RNA intermediate for transposition. Class II or DNA transposons do not require a RNA intermediate and typical DNA TEs insert into new locations by a cut and paste mechanism [20,22].

TEs have been further classified into superfamilies. The long terminal repeats (LTRs) are made up of *Gypsy*, and *Copia*. DNA transposons are classified into at least 17 superfamilies and encode a DDE/D domain found in all transposases [23]. The most common TEs found in fungal genomes are *Gypsy*, *Copia*, and *Tc1-Mariner* [24,25]. Functional LTR transposons have open reading frames (ORFs) coding for *gag*, *pol*, and *env* (in endogenous retroviruses). The pol ORF codes for reverse transcriptase (with integrase, reverse transcriptase, and RNAse H protein coding domains) and an aspartic protease required for polypeptide maturation. The entire element is flanked by LTRs. Many LTR transposons have lost one or more functional ORFs required for transposition and thereby lose the ability to replicate autonomously. Long interspersed nuclear repeats (LINE) are another type of Class I transposons that usually possess polyA-tails but are not flanked by LTRs. Intact LINE retrotransposons usually encode two ORFs; the first with a RNA binding domain and the second with endonuclease and reverse transcriptase domains. In fungi, the two most common Class II TE are *Tc/Mar* and *hAT* superfamilies [26,27]. These are comprised of a DDE transposase with a characteristic transposase dimerization domain flanked by very short terminal inverted repeats [26,28].

Owing to the importance of this fungal pathogen, *Coccidioides* spp. were one of the first dimorphic fungi to have a sequenced genome [3,29]. Sequencing of *C. immitis* and *C. posadasii* genomes by Sanger sequencing technologies produced relatively complete chromosome-scale genome maps. The assembly of *C. immitis* strain RS is considered more complete than *C. posadasii* C735 based on the scaffolds of assembly contigs [3]. Additional lower coverage genome sequencing of multiple strains of *C. immitis* and *C. posadasii* has been performed to better examine the diversity of these species. In two studies the total repetitive content of multiple isolates of *C. immitis* and *C. posadasii* was estimated to be from 8–21% of the genome, with many individual TE insertions specific to one assembly [3,25].

Due to differences in genome annotation applied to the initial two *Coccidioides* spp. genomes, TEs were handled and masked out with different stringencies so the TE content of the *C. immitis* as compared to *C. posadasii* cannot be directly compared from the published annotation. In addition, databases and methods for TE detection and annotation have improved, so a common and consistent bioinformatics approach in each species is necessary to make comparisons. One study focused on LTR TE’s in fungi including *Coccidioides* spp. explored their content from a genome-ecology perspective and found that the genomic proportion of Class II TEs was highly variable across the Ascomycota [25]. Comparison of *C. immitis* and *C. posadasii* found a relatively similar TE content while a close relative, the nonpathogenic *Uncinocarpus reesii*, had a much lower TE content. One major component of the TE content in the *Coccidioides* spp. genomes are two closely-related families of *Gypsy* transposons that were found in isolates of both *C. immitis* and *C. posadasii*. This suggests that these transposons have been retained in the *Coccidioides* spp. genomes since the species diverged 5 mYA ago.

We have characterized the TEs in *Coccidioides* spp. and investigated the relationship of TEs and nearby genes in *Coccidioides* spp. taking advantage of the genomic and transcriptomic resources of this closely related pair of species. As the TE content likely varies more than the gene content, these data provide an opportunity to compare transcript expression of a set of orthologous gene loci differing by their proximity to a transposable element. The currently available transcription studies were performed with the RS strain of *C. immitis* and the C735 strain of *C. posadasii*, so we have focused on these two strains.

## 2. Materials and Methods 

### 2.1. Genomes

The genomes and annotations of *C. immitis* RS strain and *C. posadasii* C735 strain were obtained from FungiDB [30,31]. TE annotation was performed using RepeatMasker [32] which identified TEs based on the RepBase reference library and REPET to support de novo TE identification [33]. The results of these annotation and supporting pipeline scripts are provided in the Github repository [34].

### 2.2. Analysis tools

Calculation of GC content and ORF prediction was done using tools in Galaxy [35]. Prediction of polyprotein domains was done by blastx analysis of *Gypsy* TEs against the Swiss-Prot database. A BLASTX hit of <10^−8^ to a polyprotein was considered positive. Phylogenetic relationships of *Gypsy* domains were assessed by performing multiple alignments using Clustal X; the relations were displayed using the Interactive Tree of Life tools [36]. TEs were mapped to contigs using igv [37].

Identification of loci within 1 kB of TEs and Gene Ontology term enrichment identification of genes in *C. immits* or *C. posadasii* were performed within FungiDB. Tables of normalized gene expression values of protein coding genes based on RNAseq of *C. immitis* and *C. posadasii* mycelia and spherules from prior studies were also obtained from the FungiDB presentation of Whiston et al. results [9]. The data are expressed as log_2_ transformed unique fragments per kilobase of transcript per million mapped reads (FKPM).

Statistical comparisons of the association of gene expression and presence of TEs was performed with ANOVA and the Dun’s post-test in R. Reported *p*-values are corrected by the Bonferroni adjustment for multiple testing. Scripts are provided in the Github repository associated with this publication [34].

## 3. Results

The total repetitive DNA content of *C. immitis* and *C. posadasii* DNA identified in this study is 17–19% of the genome (Table 1), very similar to the previously reported values for these genomes [3,25,29]. All the identified repetitive elements are listed in Table S1. About 70% of the repetitive DNA is encoded by Class I LTR elements, Class II elements and LINE elements. *Gypsy* is the most common type of TE found in both species, and is typically the most abundant in many fungi [24,25].

The relative proportions of TE and total repetitive DNA are similar in *C. immitis* and *C. posadasii* (Table 1). Notable differences include more *hAT* elements and fewer *TcMar* elements in *C. immitis* as compared to *C. posadasii*. Many TEs contain ORFs longer than 300 nucleotides; the ratio of ORF to TE was lowest in *TcMar* and highest in *Gypsy*. Some *Gypsys* contained more than one ORF. The GC content of DNA coding for TEs is 28–39%, markedly lower than the average GC content for the total *Coccidioides* spp. genomes (46%), which is consistent with a RIP mechanism as has been suggested by others [3,38].

We focused on *Gypsy* TEs for a more detailed analysis. The presence of domains was analyzed by BLASTX searching the Swiss-Prot database for homolog to LTR polyproteins (Evalue ≤ 10^−8^). Using this approach, 338 of 1204 (28%) *C. immitis Gypsy* and 22% of *C. posadasii* TEs contain regions homolgous to a polyprotein domain (Table 2). In both species, *Gypsy* elements lacking a polyprotein domain were more likely to be within 1 kB of a locus, suggesting that *Gypsy* TEs lacking polyprotein domains were found in more gene-rich regions than those with polyprotein domains.

**Figure 1:**
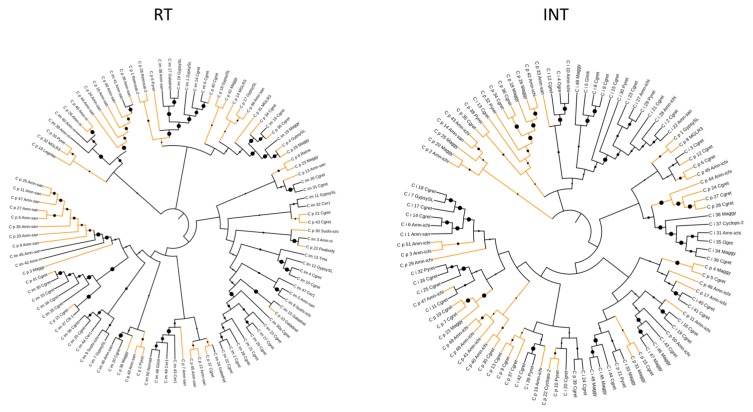
Phylogenetic relationship between RT and INT domains in *C. immitis* and *C. posadasii*. The black lines represent *C. immitis Gypsy* RT and INT domains; the orange lines represent *C. posadasii Gypsy* RT and INT domains. The size of the black dot represents the bootstrap value.

Reverse transcriptase (RT) and integrase (INT) domains were aligned using Clustal X to investigate their evolution and a Bootstap N-J tree inferred in order to investigate their evolution. The trees representing the relationships of RT and INT domains of the *Gypsy* polyproteins from *C. immitis* and *C. posadasii* are shown in Figure 1. The two trees show that for the most part RT and INT domains of the two species are more closely related within the species than between species. The species-specific divergence suggests that transposition has occurred after species diverged, followed by independent evolution of the domains in each of the *Coccidioides* lineages. It has been noted in previous comparative genomics studies of multiple *Coccidioides* strains that the genomes differed by individual insertions, indicating that transposable elements have been active in multiple strains [3,25].

**Figure 2:**
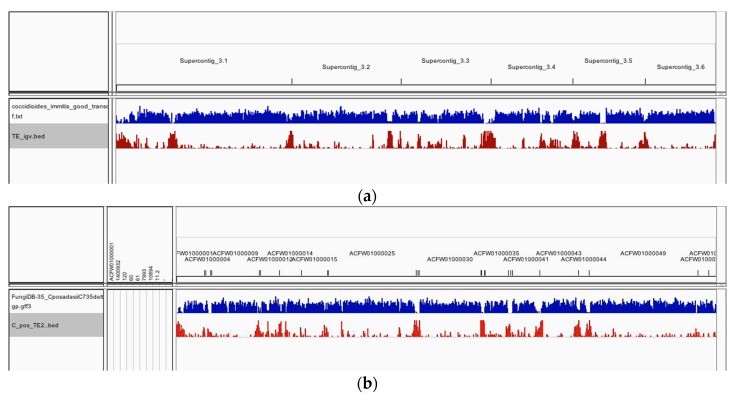
The genomic distribution of protein-coding loci (blue track) and the TEs (red track) in *C. immitis* (**a**) and *C. posadasii* (**b**) assemblies. These data show an inverse relationship between predicted genes and TEs and the tendency of TEs to cluster at the ends of contigs, reflecting both assembly difficulties and potential preferential accumulation regions. The *C. immitis* genome is mapped on six contigs; *C. posadasii* on 20.

**Figure 3:**
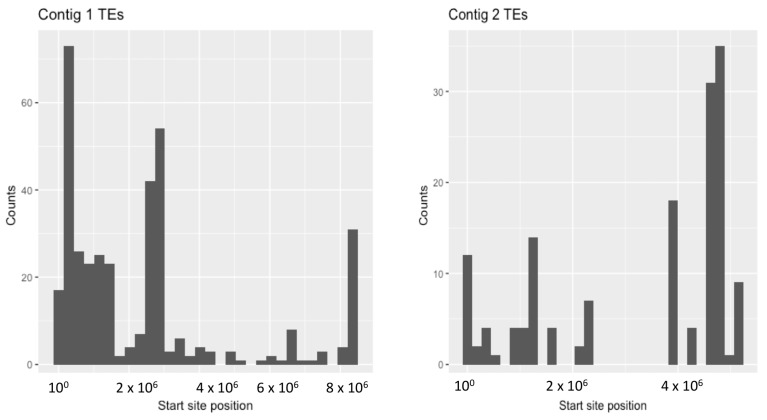
Histogram showing the frequency of TEs in the two largest *C. immitis* contigs. The number of *C. immitis* TEs is plotted on the Y axis and the position on contigs 1 and 2 is plotted on the Y axis. A bin width of 5% of the contig length was used.

The distribution of TEs along the *C. immitis* and *C. posadasii* contigs in shown in Figure 2. There is a tendency in both species for TEs to occur in clusters enriched at the ends of contigs. The clusters are shown more dramatically as histograms of the number of *C. immitis* TEs plotted against position on contigs 1 and 2 (bins of 5% length) (Figure 3). The mapping pattern of *C. immitis* and *C. posadasii* TEs is similar, although many *C. posadasii* TEs are found on very short contigs, which likely reflects the difficulty in assembling repetitive DNA or a less robust *C. posadasii* assembly. DNA and LTR TEs appear to be randomly distributed within the clusters. Overlapping TEs are relatively uncommon; only 273 (13%) of the *C. immitis* and 190 (9%) of the *C. posadasii* TEs are nested.

**Figure 4:**
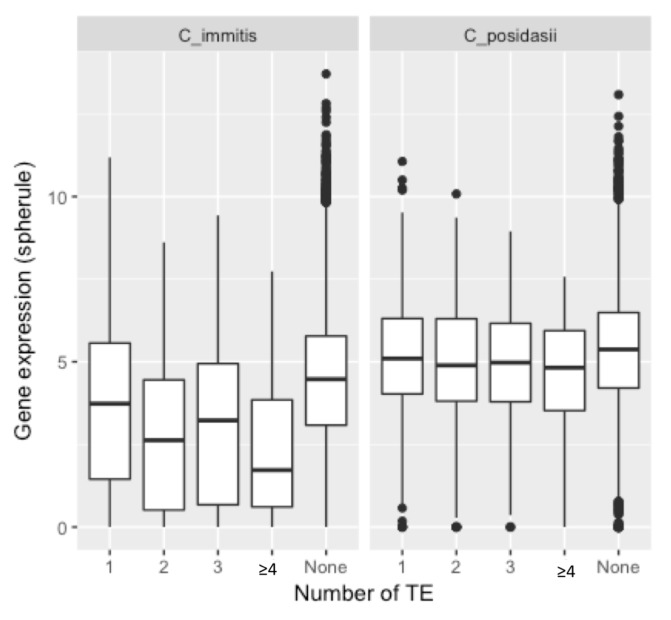
Expression (median values log_2_ FPKM) of *C. immitis* and *C. posadasii* loci within 1 kB of one or more TE. The *p*-values for gene expression of all *C. immitis* groups flanked by at least one TE compared to control expression levels (“None”) are less than 1 × 10^−4^. The *p*-values of *C. posadasii* gene expression of genes flanked by at least two TEs is 7 × 10^−3^ compared to the control (“None”). The remaining *C. posadasii* gene expression values are not significantly lower than the control value.

There are a total of 902 *C. immitis* protein-encoding loci flanked by a TE within 1 kB. Many of these loci are in the lowest quartile of expression compared to all loci. The mean expression level of *C. immitis* genes within 1 kB of a TE is significantly less (3.60 FPKM) than the level of expression in controls (4.29 FPKM) (*p* < 1 × 10^−4^) (Figure 4). The level of expression for the spherule stage is shown and essentially identical results were found when exploring the expression in hyphae. Expression of *C. immitis* genes within 1 kB of four or more TEs was very low (*p* < 1 × 10^−15^). In *C. posadasii*, 773 loci were associated with TEs. *C. posadasii* loci associated with multiple TEs were also poorly expressed (*p* < 10^−2^), but but the proportion of poorly expressed genes was much lower than in *C. immitis*.

Fifty-five *C. immitis* genes were within 1 kB of four or more TEs. Seventeen of 55 (31%) of these genes were close to the end of a contig (within 300 bp). Twenty-seven of the 55 genes had orthologs in *C. posadasii* and 10 of the 27 (37%) *C. posadasii* homologs were also close to the end of a contig. 

Twenty-six of the 27 *C. posadasii* orthologs are within 1 kB of at least one TE and 17/27 are within 1 kB of four or more TEs (Table 3). To address this question further, 295 *C. posadasii* orthologs of the 571 *C. immitis* loci flanked by a *Gypsy* TE were identified. One-hundred eighty-one (61%) of these *C. posadasii* genes were also within 1 kB of a TE. Since only 772 of a total of 7255 (11%) of *C. posadasii* loci were associated with TEs, these data suggest that many more orthologous loci are also associated with TE than would be expected by chance (chi-square < 10^−10^).

**Figure 5:**
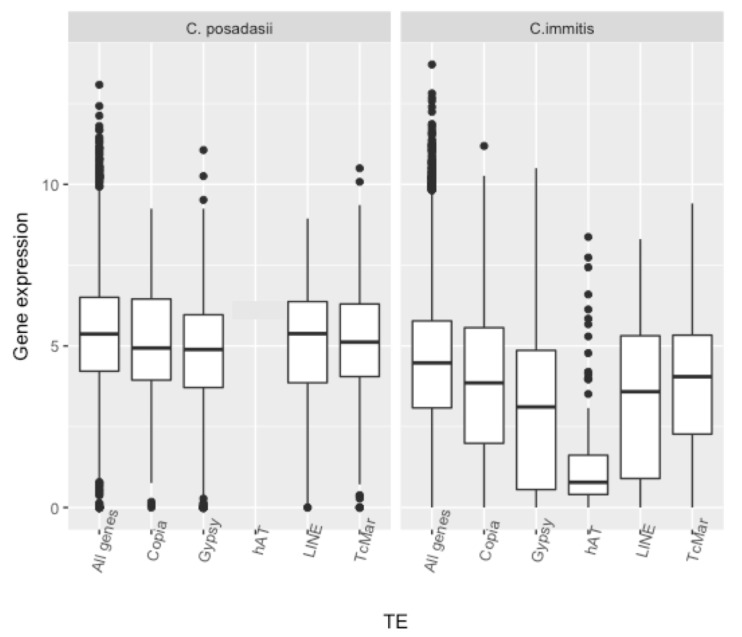
Mean expression values (log_2_ FPKM) of *C. immitis* and *C. posadasii* loci within 1 kB of TE superfamilies compared to all loci. The *p*-values for *C. posadasii* genes associated with *Gypsy* were 5 × 10^−3^ and all others were not significant. The *p*-values for *C. immitis* genes associated with any TE superfamily were all ≤1 × 10^−4^; *p*-values for *C. immitis* genes associated with *Gypsy* or *hAT* TEs were ≤1 × 10^−10^.

The association of different superfamilies of *C. immitis* and *C. posadasii* TEs with the level of expression of nearby loci is shown in Figure 5. In *C. immitis*, loci within 1 kB of *Gypsy* and *hAT* TEs are the most poorly expressed. Loci within 1 kB of *TcMar*, *Copia* or LINE TEs are also expressed at a lower level than the average for all loci. TEs did not have an effect on the relative expression of genes in hyphae compared to spherules. The association of poor gene expression with TEs was much less impressive in *C. posadasii*. *Gypsy* TEs were associated with relatively poorly expressed loci (*p* < 5 × 10^−3^) but none of the other superfamilies were. The relationship of *hAT* to gene expression in *C. posadasii* could not be determined because only eight loci were within 1 kB of this TE.

In *C. immitis*, the relationship of locus expression to the relative position of the TEs depends upon the TE superfamily (Table 4). Genes with a 5′ flanking *Gypsy* or contained within a *Gypsy* TEs are expressed at a lower level than 3′ flanking TE. Genes within 1 kB of hAT TEs also have low expression levels regardless of relative location. However, the relative location of genes to *TcMar* or *Copia* TE’s does not seem to influence expression. In *C. posadasii*, the flanking direction of the *Gypsy* TE to the locus did not appear to be correlated with the level of expression.

The FungiDB Gene Ontology Enrichment tool was used to test whether there was a bias in the predicted function of TE-associated genes. TE-associated genes from both *C. immitis* and *C. posadasii*, were enriched for phosphorylation and protein phosphorylation Gene Ontology (GO) terms (Table 5). The TE-associated protein phosphorylation genes have a similar expression level in hyphae and spherules.

Forty-one TE-associated *C. immitis* genes were classified with the protein phosphorylation functional GO term 0006468 (listed in Table S2). These genes were found dispersed over all contigs. Fourteen were described as CMGC or CMGC/SRPK kinases, nine as serine/threonine protein kinases, 11 as hypothetical proteins, and the remainder as other kinases. Forty-two *C. immitis* genes associated with TE were classified with the phosphorylation functional GO term 0016310 (Table S2). Twenty-six of these were also annotated with the protein phosphorylation GO term. Thirteen of the 42 genes were classified as CMGC or CMGC/SRPK kinases, nine as serine/threonine kinases, 11 as hypothetical proteins, and the remainder as other kinases.

## 4. Discussion

This analysis of the genomes of *Coccidioides* spp. finds that TEs comprise 17–19% of the genome, a similar proportion of repetitive DNA found in previous studies [3,25,29]. *Coccidioides* spp. like all fungi, contains both Type I retrotransposons and Type II DNA transposons. The most common TE identified was *Gypsy*, as has been previously reported [3,25]. All TE superfamilies contain some open reading frames >300 nt; *Gypsy* TEs contains the highest proportion of ORFs and *TcMar* the lowest. Only 20–30% of *Gypsy* TEs contain ORFs with polyprotein domains indicating that most are non-autonomous copies that have extensive modification. Non-autonoumous *Gypsy* TEs lacking polyprotein domains were more frequently found within 1 kB of protein coding genes, suggesting that active TEs may not be tolerated in actively transcribed regions, although the mechanism for exclusion or genome defense of genic regions requires further exploration. Phylogenetic analysis of *Gypsy* reverse transcriptase and integrase showed that the domains tended to cluster within species, but with a significant amount of mixing between species. The tendency for domains to cluster within species could be due to transposition of TEs after the species diverged, as others have reported [3,25]. Presumably, the domains that are closely related between species reflect TEs that were inserted before the species diverged. All TEs have a low GC content, which is consistent with repeat-induced point mutation as previous studies have suggested [3,38].

TEs in a variety of organisms tend to occur in clusters that are predominantly found at the telomeres or centromeres [39,40]. Many of the *Coccidioides* spp. TEs are found in clusters that contain both Class I and Class II TEs. Only a few of the TEs are nested suggesting that transposition may not be ongoing into targeted locations. Clusters of *Coccidioides* spp. TEs tend to be found primarily at the ends of contigs, as has been observed in some other fungal genomes [41,42,43]. Since the location of the centromeres and telomeres in *Coccidioides* spp. are currently undescribed, it is impossible to comment on association of TEs within or proximal to those structures.

We found that *C. posadasii* genes that are orthologs of *C. immitis Gypsy*-associated genes also tend to be TE-associated. *C. posadasii* orthologs of *C. immitis* genes flanked by multiple TEs tend to be associated with multiple TEs too. This suggests that insertion of TEs may have preferentially occurred near certain genes. In other organisms LTR transposons have been found to preferentially integrate into regions near a subset of genes [44].

Our analyses point to a common trend of reduced gene expression of loci with nearby TEs, especially in *C. immitis*. Many genes within 1 kB of a TE are relatively poorly expressed. This effect is most striking if more than one TE is near a gene. The type of TE has an influence as well, with genes near *hAT* or a *Gypsy* TEs showing the lowest average expression. The relative position of *C. immitis* genes to TE is correlated with lower expression if the TE is a *Gypsy* but not other types of TEs. Furthermore, the function of genes associated with TEs is non-random as there is an enrichment of protein phosphorylation and phosphorylation functional categories.

*C. posadasii* TE-associated genes are expressed at a more typical level. On average, the expression of genes near multiple TEs was modestly lower than all genes. Comparing the effect of different TE superfamilies, only *Gypsy C. posadasii* elements showed a significant correlation with reduced gene expression. Only eight genes are within 1 kB of a *hAT* TE in *C. posadasii*, limiting the resolution and sufficient observations to test if this class of TEs was also associated with poor expression of loci. Although the mean level of expression of genes associated with TEs is not as low in *C. posadasii* as in *C. immitis*, we do not know why. The two species have diverged significantly, which may play a role in the difference.

The significance of the association of multiple TEs or *hAT* and *Gypsy* TEs with poorly expressed protein-encoding loci in *C. immitis* is unclear. It it also not clear what mechanism accounts for this association. One possibility is that some superfamilies of TEs tend to co-localize with poorly-expressed genes. Another is that some TEs suppress gene expression. This data does not distinquish between those possibilities.

TEs have been documented to influence gene expression in many of organisms [33,45,46,47], often modulating genes that code for stress responses and host defenses [17,48]. There are a variety of mechanisms by which TE’s can influence gene expression. The most direct mechanism would be inactivation or enhancement of promoter activity influencing gene expression [49]. TE insertions in the UTR could also effect on mRNA stability [16]. DNA methylation can specifically target repetitive regions and may be an important mechanism for gene silencing [50,51,52]. Although there have been no published studies of DNA methylation in *Coccidioides* spp., both *C. immitis* and *C. posadasii* have transcribed homologs of *Dim-1* cytosine methylase, supporting the possibility that DNA methylation silencing could provide transposon control. DNA methylation targeting TEs that spreads to neighboring genes could explain the observed reduced expression of genes proximal to TEs. TEs are associated with heterochromatin formation, which could decrease expression of associated loci [47,53]. Inhibitory small RNAs are another mechanism that could play a role in targeting transcript repression [54]. There is no direct assessment of RNAi or small RNA activity in *Coccidioides* spp., although both species contain genes coding for RNAi machinery namely RNA-dependent RNA polymerase, Argonaute and Dicer proteins. It is possible that more than one suppressive mechanism is playing a role for one TE or that different TEs suppress gene activity by different mechanisms.

In contrast to the difference in gene TE-associated gene silencing in *C. immitis* and *C. posadasii*, TEs in both species are associated with genes coding for phosphorylation, including protein phosphorylation. It is interesting that many protein kinase genes are down-regulated as *C. immitis* hyphae differentiate into spherules [11]. The link between TEs and genes coding for phosphorylation in both *C. immitis* and *C. posadasii* is consistent with the hypothesis that this association may have occurred before the divergence of the two species and that there may be some evolutionary pressure to maintain that association. A number of TEs in other organisms have been found to preferentially occur at specific sites within the genome. Some of the most common genes associated with TEs code for transfer RNA, ribosomal RNA, silent mating genes and genes involved in the formation of heterochromatin [44,55,56]. Some TEs have also been found to preferentially insert near protein phosphorylation genes [57].

In summary, this is a report characterizing the transposable elements in two *Coccidioides* spp. An association of some TEs with poorly expressed protein-encoding genes in *C. imitis* and a genomic location association of TEs with phosphorylation enzymes in both species of *Coccidioides* has been observed. This is a bioinformatic study characterizing TEs and their association with genomic and transcriptomic data. It is our hope that the data will suggest hypotheses about gene regulation in *Coccidioides* spp. for future experimental testing.

## Figures and Tables

**Table 1:** Predicted TEs in *Coccidioides* spp.

	*C. immitis*				*C. posadasii*			
Type	Number	Mean Length	ORFs > 300 nt (%)	GC Content (%)	Number	Mean length	ORF > 300 nt (%)	GC Content (%)
DNA/*TcMar*	286	884	113 (40)	37	575	991	456 (79)	36
-Ant1	63				31			
-Fot1	134				430			
-Pogo	27				29			
-Tc1	66				83			
DNA/*hAT*	100	2416	80 (80)	36	37	1232	15 (40)	31
LTR/*Gypsy*	1204	2089	1557 (130)	33	1199	2046	1387 (116)	34
LTR/*Copia*	287	1351	151 (52)	38	190	937	131 (69)	39
LINE	364	1331	239 (66)	28	225	1237	144 (64)	31

**Table 2:** Polyprotein domains in *Gypsy* TEs.

	*C. immitis*			*C. posadasii*		
	Total	Pol Domain	None	Total	Pol Domain	None
Number of TEs	1204	338 (28%)	866 (72%)	1199	260 (22%)	938 (78%)
Associated loci	571	60 (11%)	511 (89%)	341	24 (7%)	317 (91%)

**Table 3:** *C. posadasii* orthologous protein-encoding loci associated TEs.

	Number of TEs				
*C. posadasii* orthologs	≥4	3	2	1	None
27	17	2	5	4	1

**Table 4:** Relationship of gene location to TE to gene expression in *C. immitis*.

	Control	Upstream ^a^		Overlap ^b^		Downstream ^c^	
	Mean	Number	Mean	Number	Mean	Number	Mean
All genes	4.032	992		773			
*TcMar*		115	3.734	131	3.759	115	3.734
*hAT*		75	1.134	73	1.135	77	1.292
*Copia*		119	3.242	69	3.343	137	4.229
*Gypsy*		272	3.027	217	2.001	312	2.156

^a^. Gene upstream of TE; ^b^. TE overlaps gene; ^c^. Gene downstream of TE.

**Table 5:** GO enrichment analysis of genes associated with TEs.

		*C. immitisb*		*C. posadasii*	
ID	Name	Odds Ratio	*p* (Bonferroni)	Odds Ratio	*p* (Bonferroni)
GO:0016310	Phosphorylation	3.35	1.43 × 10^−7^	3.34	7.35 × 10^−7^
GO:0006468	protein phosphorylation	3.47	1.56 × 10^−7^	3.60	2.10 × 10^−6^
GO:0036211	protein modification process	2.72	3.87 × 10^−6^	2.48	2.90 × 10^−4^
GO:0006464	cellular protein modification process	2.72	3.87 × 10^−6^	2.48	2.90 × 10^−4^
GO:0006796	phosphate-containing compound metabolic process	2.45	1.17 × 10^−5^	2.07	4.41 × 10^−3^
GO:0006793	phosphorus metabolic process	2.43	1.36 × 10^−5^	2.05	5.00 × 10^−3^

## Supplementary Materials

The following are available online at www.mdpi.com/2309-608X/4/1/13/s1. Table S1. *C. immitis* and *C. posadasii* repeats. Table S2. *C. immitis* loci with protein phosphorylation or phosphorylation functional GO terms.
